# Genome-wide identification and characterization of UDP-glucose dehydrogenase family genes in moso bamboo and functional analysis of *PeUGDH4* in hemicellulose synthesis

**DOI:** 10.1038/s41598-020-67227-8

**Published:** 2020-06-23

**Authors:** Ying Yang, Lan Kang, Ruihua Wu, Yuzhen Chen, Cunfu Lu

**Affiliations:** 1 Beijing Advanced Innovation Center for Tree Breeding by Molecular Design, Beijing Forestry University, Beijing, 100083 China; 2 College of Biological Sciences and Technology, Beijing Forestry University, Beijing, 100083 China

**Keywords:** Molecular biology, Physiology

## Abstract

Uridine diphosphate glucose dehydrogenases (UGDHs) are critical for synthesizing many nucleotide sugars and help promote the carbohydrate metabolism related to cell wall synthesis. In plants, UGDHs are encoded by a small gene family. Genome-wide analyses of these genes have been conducted in *Glycine max* and *Arabidopsis thaliana*, however, the *UGDH* gene family has not been comprehensively and systematically investigated in moso bamboo (*Phyllostachys edulis*), which is a special woody grass monocotyledonous species. In this study, we identified nine putative *PeUGDH* genes. Furthermore, analysis of gene duplication events and divergences revealed that the expansion of the *PeUGDH* family was mainly due to segmental and tandem duplications approximately 4.76–83.16 million years ago. An examination of tissue-specific *PeUGDH* expression indicated that more than 77% of the genes were predominantly expressed in the stem. Based on relative expression levels among *PeUGDH* members in different tissues in moso bamboo, *PeUGDH4* was selected for detailed analysis. The results of subcellular localization indicated that PeUGDH4-GFP fusion proteins was observed to be localized in the cytoplasm. The ectopic overexpression of *PeUGDH4* in *Arabidopsis* significantly increased the contents of hemicellulose and soluble sugar, suggesting that *PeUGDH4* acts as a key enzyme involved in bamboo cell wall synthesis.

## Introduction

The presence of a cell wall, which provides rigidity and flexibility, is one of the main characteristics that differentiates plant cells from animal cells. The cell wall, comprised mainly of complex polysaccharides (cellulose, hemicellulose, and pectin) and some structural proteins, is critical for plant growth^1–3^. Additionally, UDP-glucose (UDP-Glc) is the chief form of activated sugar, representing a branch point of glucose metabolism^4^ and a major substrate in many glycosylation reactions. For example, UDP-Glc is the substrate used by sucrose phosphate synthase to synthetize sucrose-6-phosphate in the cytosol. Moreover, in plastids, UDP-Glc is essential for the direct or indirect (via ADP-glucose) production of starch^5^. Therefore, UDP-Glc is indispensable for the synthesis of sucrose, cellulose, and callose, which contribute to cell wall formation.

The UDP-glucose dehydrogenases (UGDHs) are involved in the synthesis of matrix polysaccharides and can convert UDP-glucose into UDP-glucuronate (UDP-GlcA) and produce two NAD^+^ molecules. Furthermore, UDP-GlcA is not only the intermediate pivot of polysaccharide metabolism, it is also an important glucuronic acid donor during cell wall polysaccharide synthesis. Previous studies revealed that UDP-GlcA continues to be glycosylated to form UDP-galacturonic acid, UDP-xylose, UDP-apiose, and other nucleoside sugars participating in the biosynthesis of hemicellulose and pectin, which represent over half of the cell wall biomass in *Arabidopsis thaliana* leaves^6–8^. Because these multi-step glycosylation reactions are irreversible, UGDHs may be crucial for cell wall formation^9^.

The first UGDH was identified in the bovine liver in 1954^10^ and was subsequently purified in 1969^11^. The *Glycine max GmUGDH* gene cloned 30 years later^12^ was the first confirmed plant *UGDH* gene. Analyses of the 10 *UGDH* genes that have since been detected in the *G. max* genome database revealed the *GmUGDH* genes are highly conserved and encode a region typical of UDP-glucose/GDP-mannose dehydrogenase family members^13^. Two highly similar *UGDH* genes were identified in maize^14^ based on a search of the GnpSeq Genoplante database. In *A. thaliana*, AtUGDH2 (AT3g29360) and AtUGDH3 (At5g15490) are the main enzymes catalyzing the conversion of UDP-Glc to UDP-GlcA^15^.

Bamboo species form one of the most important forest resources with extremely strong and flexible cell walls^16,17^^.^ Moso bamboo (*Phyllostachys edulis*) is the most cultivated and economically valuable bamboo species in China^18–21^. Previous research on moso bamboo indicated its tensile strength, which is approximately double that of normal wood, combined with high flexibility and ductility prevent the plants from breaking under snow pressure^22^. Previous findings have confirmed that *UGDH* genes contribute to polysaccharide metabolism and cell wall biosynthesis. However, the moso bamboo *PeUGDH* genes remain relatively uncharacterized. In this study, we identified nine putative *PeUGDH* genes and investigated phylogenetic relationships, gene and protein structures, chromosomal localization, evolution and divergence patterns and expression levels in diverse tissues. The overexpression of *PeUGDH4* in *Arabidopsis* resulted in an obvious increase in hemicellulose synthesis. This study will serve as a useful reference for further functional analyses of candidate genes involved in bamboo cell wall development and for future molecular studies of *UGDH* genes in related plant species.

## Results

### Database search for moso bamboo PeUGDH genes

To identify the *P. edulis* UGDH family members, *O. sativa* UGDH amino acid sequences were used as queries for a BLAST search of a bamboo database. Eleven candidate PeUGDHs were identified and subsequent analyses with the Pfam database and NCBI CD-search revealed that nine of the sequences contained three expected conserved domains, UDPG-MGDP-dh-N (Pfam: 03721), UDPG-MGDP-dh-C (Pfam: 03720), and UDPG-MGDP-dh (Pfam: 00984). Chromosome mapping results indicated the *PeUGDH* genes were distributed on three moso bamboo chromosomes (10, 15, and 21). Details regarding the *PeUGDH* genes, including gene ID, chromosomal location, and other basic information are listed in Table 1. All PeUGDH*s* were predicted to be stable, meaning they were equipped with the ability to resist the interference of various factors and maintain their own vitality^23^.

**Table 1:** Molecular characterization of nine predicted moso bamboo *PeUGDH* genes.

Gene ID	Location	CDS length (bp)	Protein				
			Size (aa)	MW (Da)	pI	Atomic composition	Instability
PH02Gene21682.t1	21:14393166:14394608	1443	480	52854.81	6.16	C_2373_H_3750_N_628_O_698_S_19_	Stable
PH02Gene38725.t1	15:90042098:90043540	1443	480	52859.73	5.72	C_2373_H_3745_N_623_O_703_S_19_	Stable
PH02Gene21681.t1	21:14372070:14373512	1443	480	52891.80	5.85	C_2372_H_3745_N_625_O_702_S_20_	Stable
PH02Gene29082.t1	21:98887575:98892948	1716	571	62869.57	8.07	C_2813_H_4487_N_755_O_826_S_24_	Stable
PH02Gene19803.t1	15:64056396:64057838	1443	480	52721.57	5.84	C_2365_H_3735_N_623_O_701_S_19_	Stable
PH02Gene09808.t1	21:40805665:40808401	1380	459	51065.34	5.98	C_2205_H_3478_N_608_O_652_S_19_	Stable
PH02Gene20841.t1	15:29407275:29408696	1305	434	48078.86	5.48	C_2155_H_3380_N_576_O_642_S_14_	Stable
PH02Gene47615.t1	10:21633092:21634519	1332	443	48077.30	8.18	C_2137_H_3406_N_594_O_628_S_19_	Stable
PH02Gene47617.t1	10:21682441:21683859	1284	427	46233.09	8.75	C_2058_H_3280_N_578_O_602_S_15_	Stable

### Multiple sequence alignment and analysis of PeUGDH proteins with Prosite

**Figure 1:**
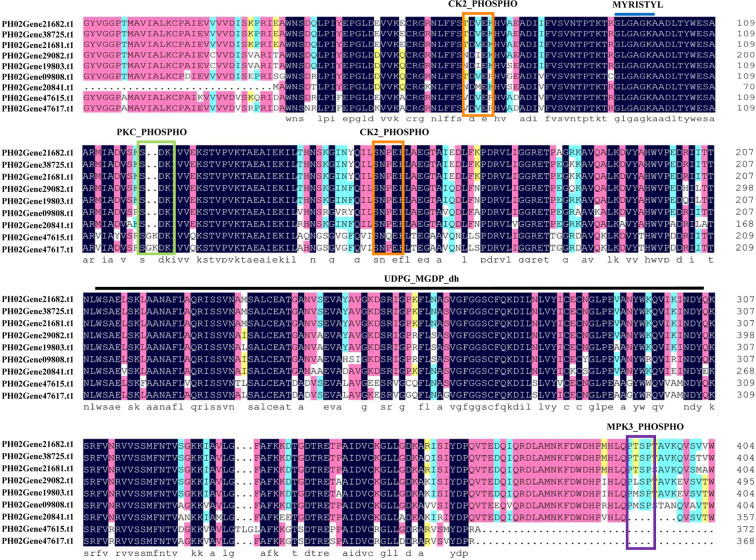
Alignment of multiple moso bamboo PeUGDH proteins with the DNAMAN software and identification of functional sites with the Prosite database (https://prosite.expasy.org/).

As shown in Fig. 1, **t**he alignment of PeUGDH sequences confirmed the proteins contained a highly conserved UDP-glucose/GDP-mannose dehydrogenase family binding region and two other core domains, UDPG_MGDP_dh_N and UDPG_MGDP_dh, which may be closely related to glycometabolism and the regulation of hemicellulose synthesis. Prosite was used to analyze additional functional sites encoded by moso bamboo *PeUGDH* genes. The results indicated that most PeUGDHs contained some phosphorylation sites, including the casein kinase II phosphorylation site (CK2_PHOSPHO_SITE), the cAMP- and cGMP-dependent protein kinase phosphorylation site (CAMP_PHOSPHO_SITE), the protein kinase C phosphorylation site (PKC_PHOSPHO_SITE), and the mitogen-activated protein kinase 3 phosphorylation site (MPK3_PHOSPHO_SITE). These sequences were highly conserved and related to the kinase phosphorylation associated with glycometabolism. Moreover, the PeUGDHs also comprised N-myristoylation sites involved in important lipid modifications influencing cell growth and signal transduction regulation.

### Phylogenetic analysis of the *PeUGDH* genes

**Figure 2:**
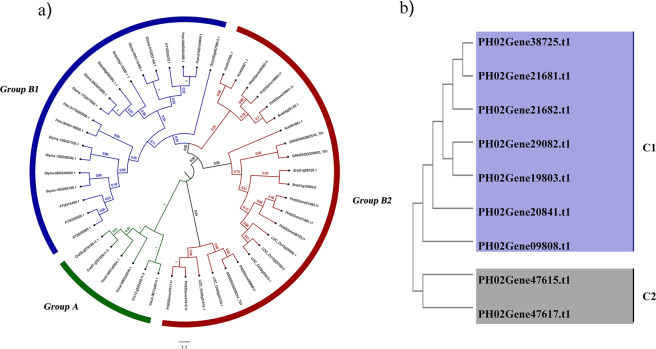
Phylogenetic analysis of UGDH proteins. (**a**) The sequences for 10 species were downloaded from the Phytozome database, whereas the PeUGDHs sequences were derived based on the moso bamboo genome. The phylogenetic tree was constructed according to the maximum likelihood method (1,000 bootstrap replicates of the MEGA 7.0 software). (**b)** Phylogenetic relationships among nine PeUGDH proteins determined with the maximum likelihood method of MEGA 7.

To investigate the origins and evolution of UGDHs among various plant species, we constructed a maximum likelihood phylogenetic tree comprising 48 full-length UGDH protein sequences from diverse plant species (Fig. 2a). All 48 UGDHs were classified into one of three groups (A, B1, and B2). The UGDHs in Group A were from aquatic algae, including *V. carteri* and *C. reinhardtii*, and are highly conserved in plants. In contrast, the other UGDHs (from terrestrial plants) were divided into Group B, with the UGDHs from dicotyledons (e.g., *A. thaliana*, *G. max*, and *P. trichocarpa*) clustered in Group B1. The PeUGDH proteins were clustered in Group B2, which also included UGDHs from typical monocotyledons, including *O. sativa*, *Z. mays*, and *B. distachyon*. The topological structure of the phylogenetic tree reflected that the *UGDH* genes originated from a common ancestor before the differentiation of aquatic and terrestrial plants. To further analyze the phylogenetic relationships among PeUGDHs, a separate PeUGDH phylogenetic tree was constructed with only nine moso bamboo PeUGDHs, which produced results consistent with the above-mentioned clustering of PeUGDHs among 11 plant species (Fig. 2b).

### Structures, conserved motif sequences, and homology modeling of *PeUGDH* genes

**Figure 3:**
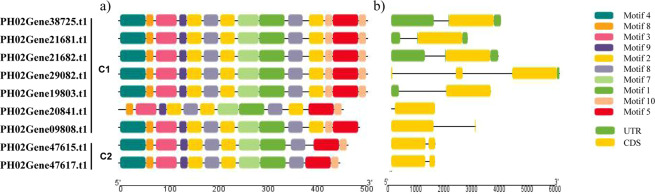
Encoded conserved motifs, and structures of nine *PeUGDH* genes. (**a)** Ten conserved motifs in PeUGDHs indicated by colored boxes. (**b)** Gene structures. Exons are presented as orange rectangles, untranslated regions (UTRs) are indicated with green rectangles, and introns are presented as black lines.

To further clarify the relationship between gene structural diversity and the specific motifs present in the nine PeUGDH proteins, the distribution of the 10 main conserved motifs in PeUGDH proteins was analyzed with the MEME online program. Moreover, ten MEME motif sequences were referred in Table S2. Similar to the results of the gene structural analysis, PeUGDHs in the same category were similar regarding the number and order of the identified conserved motifs (Fig. 3a), implying the PeUGDHs in the same category may be functionally similar. The proteins encoded by five C1 genes had all conserved motifs in the same order, whereas the proteins encoded by other genes (e.g., PH02Gene20841.t1 and PH02Gene09808.t1) lacked motif 4 or 10. Additionally, the conserved motifs in the proteins encoded by PH02Gene47615.t1 and PH02Gene47617.t1 in C2 were in the same order, but were shorter.

The structural divergence of genes is usually related to the evolution of gene families. Therefore, the structures of the moso bamboo *PeUGDH* genes were examined (Fig. 3b) to further clarify their evolution. The analysis revealed a strong link between the phylogeny and gene structure regarding the exon–intron regions and the untranslated region (UTR) length. In contrast to the other genes, PH02Gene21682.t1, PH02Gene38725.t1, and PH02Gene21681.t1 in Category 1 (C1) comprised only one exon, had relatively long upstream and downstream UTRs, and introns of a similar length. Additionally, PH02Gene29082.t1 consisted of only a downstream UTR, whereas PH02Gene19803.t1 had only an upstream UTR, but both genes were also clustered in C1. Moreover, PH02Gene47615.t1 and PH02Gene47617.t1 comprised two exons and no UTRs, with a similar exon–intron structure. Both were grouped in Category 2 (C2).

**Figure 4:**
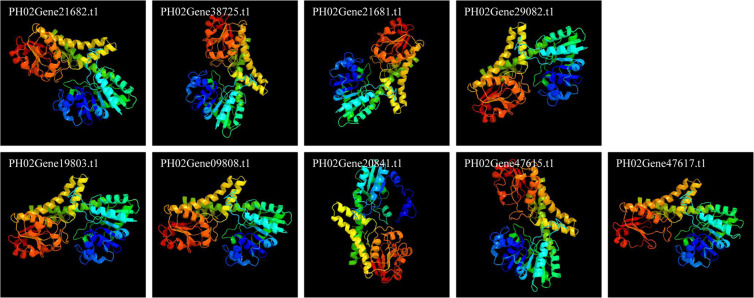
Predicted models of nine PeUGDH proteins, which were all predicted to a > 95% confidence interval via Phyre^2^ web portal (http://www.sbg.bio.ic.ac.uk/phyre2/html/page.cgi?id=index)^24^.

To elucidate the PeUGDH protein models, Phyre^2^ web portal for protein modeling, prediction and analysis (http://www.sbg.bio.ic.ac.uk/phyre2/html/page.cgi?id=index) was used^24^. The full-length PeUGDH proteins encoded by the following nine genes were modeled (100% prediction and >95% confidence): PH02Gene21682.t1, PH02Gene38725.t1, PH02Gene21681.t1, PH02Gene29082.t1, PH02Gene19803.t1, PH02Gene09808.t1, PH02Gene20841.t1, PH02Gene47615.t1, and PH02Gene47617.t1 (Fig. 4).

### The evolution and divergence patterns of *PeUGDH* genes

**Figure 5:**
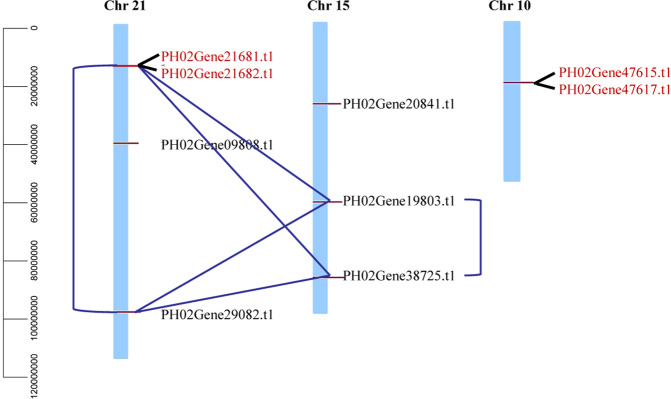
Locations of duplicated *PeUGDH* gene pairs on chromosomes 21, 15, and 10. Each *PeUGDH* was localized to a specific chromosome based on a genome annotation file. The eight pairs of segmentally duplicated genes are linked to each other by a blue line, whereas four tandemly duplicated genes are indicated in red. The scale bar represents the chromosome length.

Nine *PeUGDH* genes were localized to chromosomes 21, 15, and 10 based on the gff file from the moso bamboo genome database. The nine *PeUGDH* genes were not evenly distributed on the chromosomes (Fig. 5). Chromosome 21 had the most *PeUGDH* genes, with four, followed by chromosome 15, with three, and chromosome 10, with two. To clarify the mechanism underlying the *PeUGDH* gene family expansion, the MCScanX program was used to identify potential segmental and tandem duplication events in the moso bamboo genome. Eight segmental duplications were identified, with some *PeUGDH* genes confirmed as reciprocally duplicated genes, including PH02Gene19803.t1/PH02Gene29082.t1/PH02Gene38725.t1. These *PeUGDH* genes located on different chromosomes may have evolved from a common ancestral gene. Moreover, two tandem duplications were identified according to the physical locus of genes on chromosomes. For example, PH02Gene21681.t1 and PH02Gene21682.t1 as well as PH02Gene47615.t1 and PH02Gene47617.t1 (marked in red in Fig. 5) were located within 50 kb of each other on chromosomes 21 and 10, respectively.

To further analyze *PeUGDH* gene evolution and divergence patterns, we estimated the Ka/Ks ratios based on 10 homologous *PeUGDH* gene pairs (Table 2). The Ks value has been widely used to predict the evolutionary period of the whole genome or the period of replication events^25^. The Ks value for the 10 *PeUGDH* gene pairs varied between 0.0626 and 1.1004, indicating the replication events occurred 83.1591 million years ago (Mya) at the earliest and 4.7632 Mya at the latest. The Ka/Ks ratio was less than 1 for all gene pairs, implying the *PeUGDH* genes were mainly affected by a purification selection during evolution.

**Table 2:** *Ka/Ks* values of the homologous moso bamboo *UGDH* gene pairs.

Homologous gene pair	*Ka*	*Ks*	*Ka/Ks*	Duplication time (millions years ago)
PH02Gene21682.t1 / PH02Gene38725.t1	0.0068	0.1291	0.0527	9.8133
PH02Gene21682.t1 / PH02Gene21681.t1	0.0063	0.0790	0.0803	6.0087
PH02Gene21682.t1 / PH02Gene29082.t1	0.0493	1.0941	0.0451	83.1591
PH02Gene29082.t1 / PH02Gene21681.t1	0.0473	1.0862	0.0436	82.5576
PH02Gene38725.t1 / PH02Gene21681.t1	0.005	0.1294	0.0455	9.8348
PH02Gene47615.t1/PH02Gene47617.t1	0.0285	0.0626	0.4549	4.7632
PH02Gene19803.t1/PH02Gene38725.t1	0.0487	1.0178	0.0478	77.3540
PH02Gene19803.t1/PH02Gene21682.t1	0.0506	1.0600	0.0478	80.5637
PH02Gene38725.t1/PH02Gene29082.t1	0.0454	1.1004	0.0413	83.6320
PH02Gene21681.t1/PH02Gene19803.t1	0.0506	1.0284	0.0492	78.1620

### Expression profiles of *PeUGDH* genes and the cell wall contents in three tissues

**Figure 6:**
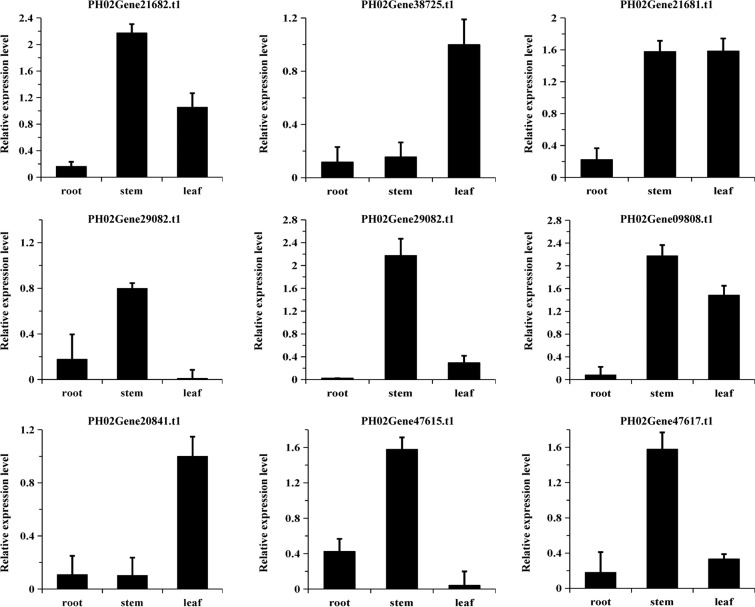
Relative *PeUGDH* expression levels in the root, stem and leaf of 100-day-old moso bamboo plants. Error bars represent SD (Standard deviation) from three independent biological replicates.

Gene expression profiles may provide relevant information for subsequent transgenic experiments and site selections for phenotypic analyses. The *PeUGDH* expression patterns were explored in a qRT-PCR assay of the transcription of nine *PeUGDH* genes in the root, stem and leaf under normal growth conditions. The qRT-PCR data (Table S3) revealed that *PeUGDH* genes were differentially expressed in all examined moso bamboo plant tissues. For example, PH02Gene21682.t1, PH02Gene21681.t1, PH02Gene19803.t1, PH02Gene09808.t1, PH02Gene47615.t1, and PH02Gene47617.t1 were highly expressed in the stem, but were expressed at much lower levels in the other tissues. Additionally, PH02Gene38725.t1 and PH02Gene20841.t1 were more highly expressed in the leaf than in the stem and root (Fig. 6).

**Figure 7:**
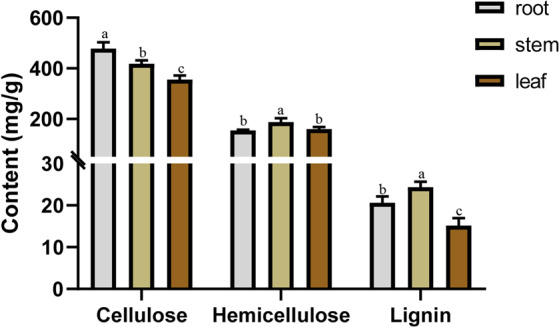
Cellulose, hemicellulose, and lignin contents in three main moso bamboo tissues. The mean values and SD were obtained from three biological replicates and a, b, c letters indicated significant differences (*P* < 0.05).

To determine the connection between *PeUGDH* expression and the abundance of cell wall components, the cellulose, hemicellulose, and lignin contents in the same tissues were measured. Lignin and hemicellulose contents were highest in the stem (Fig. 7), which was consistent with the high expression levels for most *PeUGDH* genes in the stem. However, cellulose accumulated the most in the root, followed by the stem. Therefore, we speculated that *PeUGDHs* are mainly involved in synthesizing hemicellulose and lignin, with a smaller effect on cellulose metabolism.

### Subcellular localization of PeUGDH4

**Figure 8:**
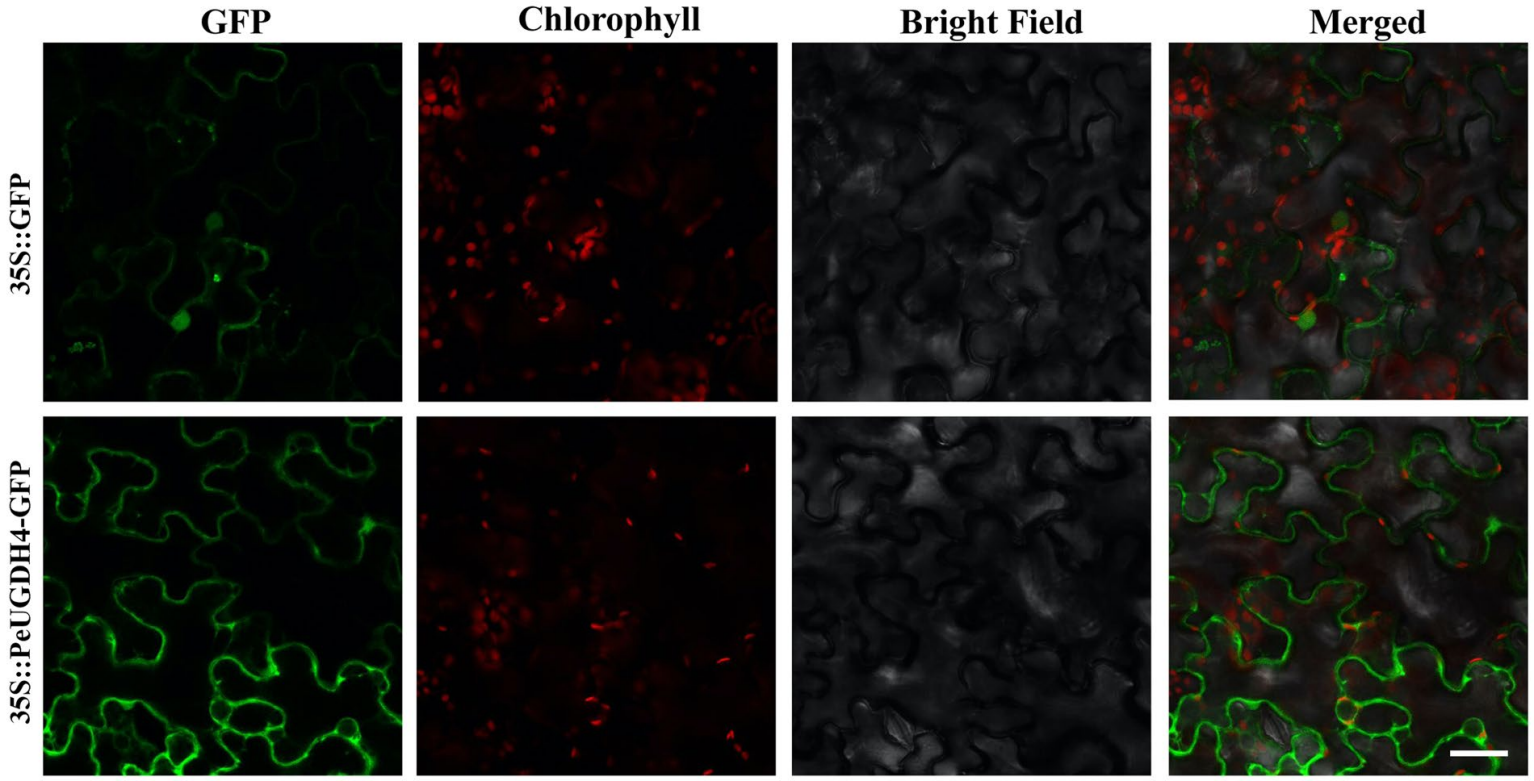
Subcellular localization of PeUGDH4-GFP fusion protein in tobacco epidermic cells. Scale = 50 μm; the full-length ORF of *PeUGDH4* was fused with GFP in pCAMBIA2300 plant expression vector drived by CaMV35S promoter (35S::PeUGDH4-GFP) and pCAMBIA2300-GFP vector (35S::GFP) was used as the positive control. Fluorescence signals are monitored by laser confocal microscopy, and the excitation light wavelengths are respectively: 488 nm for GFP and 663–738 nm for chlorophyll spontaneous fluorescence.

Based on the fact that the expression level of *PeUGDH4* was related to the lignification of bamboo shoots^26^, we considered it may be a key gene of *PeUGDHs* in the formation of cell wall. Thus, *PeUGDH4* was selected to gain further transgene research. In eukaryotes, UGDH proteins were believed to be localized in the cytoplasm^27^. To verify this, pCAMBIA2300-*PeUGDH4*-GFP gene expression vectors drived by CaMV35S promoter (Fig. S2a) was constructed and transformed into *N. tabacum*. As depicted in Fig. 8, the expression of PeUGDH4-GFP fusion protein was localized in the cytoplasm of tobacco epidermic cells, which was consistent with the analysis that no signal peptide or transmembrane domain was encoded in *PeUGDH4* gene (Fig. S3).

### *PeUGDH4* overexpression affects seedling growth and cell wall development of *Arabidopsis*

**Figure 9:**
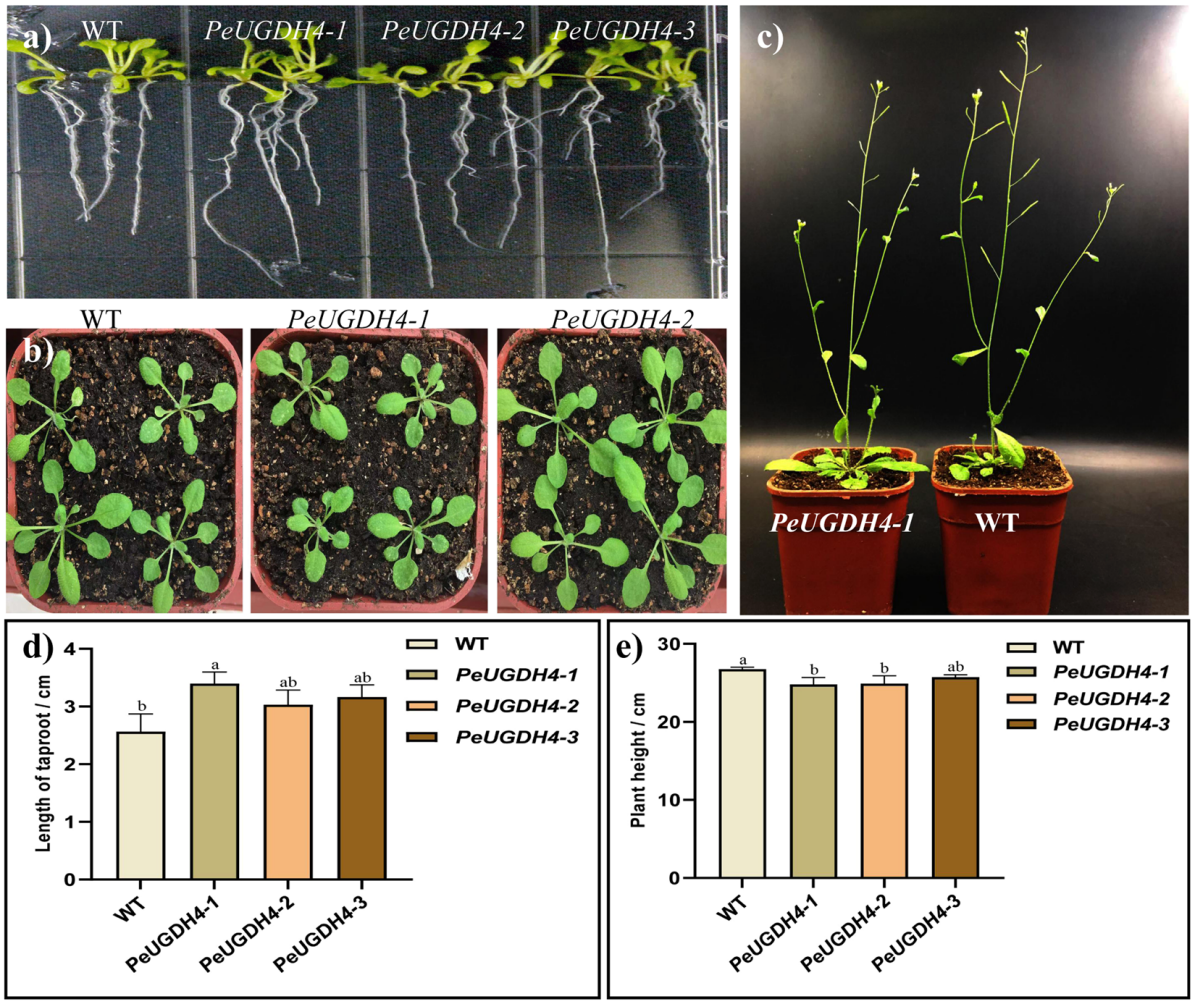
Morphology of wild type and the lines of transgenic *Arabidopsis*. (**a)** 12-day-old plants in MS medium. The three transgenic seedlings in the right side had longer taproot than wild type (left). (**b)** 27-day-old transgenic plant (right) and wild type (left). (**c)** The transgenic plants (right) were shorter than wild type (left) after 40 days. **(d)** Quantitation of longitudinal length of taproots. (**e)** Quantitation of longitudinal length of *Arabidopsis* plant height. The mean values and SD were obtained from three biological replicates with a, b, c letters indicating significant differences (*P* < 0.05).

*PeUGDH4*, driven by CaMV35S promoter, was transformed into *Arabidopsis* plants. The transgene *A. thaliana* lines named *PeUGDH4-*1, *PeUGDH4-*2 and *PeUGDH4-*3, were determined by RT-PCR (Fig. S2b,c), respectively. Compared with the wild type (WT), transgenic *A. thaliana* displayed different phenotypesat at different developmental stages (Fig. 9). The taproot length of 12-day-old transgenic lines, especially *PeUGDH4-1*, was significantly longer than that of WT plants (Fig. 9a,d). After transplanting into soil for 15 days, transgenic plants displayed no significant difference compared with the WT plants (Fig. 9b). However, after 30 d of growth in soil all transgenic *Arabidopsis* seedlings were shorter than the WT ones (Fig. 9c,e).

**Figure 10:**
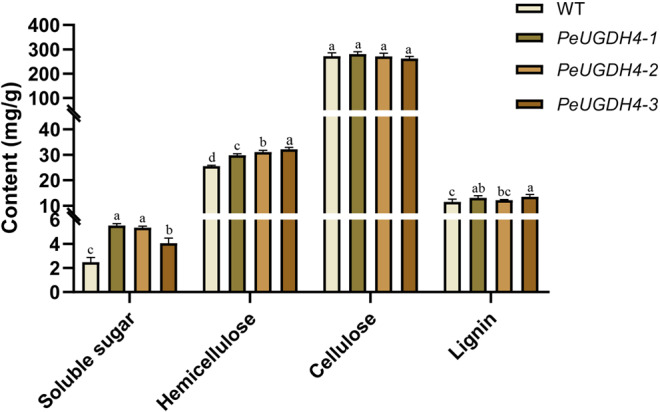
Content of soluble sugar, hemicellulose, cellulose, and lignin in 40-d-old WT and transgenic *Arabidopsis*. The mean values and SD were obtained from three biological and three technical replicates, with a, b, c, d letters indicating significant differences (*P* < 0.05).

To reveal the role of *PeUGDH4* in cell wall synthesis, the soluble sugar, hemicellulose, cellulose and lignin content in 40-day-old wild and transgenic *A. thaliana* were measured (Fig. 10). On the one hand, compared with wild type (2.48 mg), the content of soluble sugar had significantly changed in transgenic lines (4.05–5.51 mg). On the other hand, hemicellulose content in three transgenic lines was significantly higher than in the WT, but no significant difference in cellulose amount was found between transgenic *A. thaliana* and WT plants. Difference in the lignin content was evident between WT and two transgenic lines (*PeUGDH4-1* and *PeUGDH4-2* lines), but became very obvious when compared with *PeUGDH4-3* transgenic line. The above results suggested that *PeUGDH4* played a critical role in the synthesis and accumulation of soluble sugars, mainly promoting the accumulation of hemicellulose and involving in the deposition of lignin.

**Figure 11:**
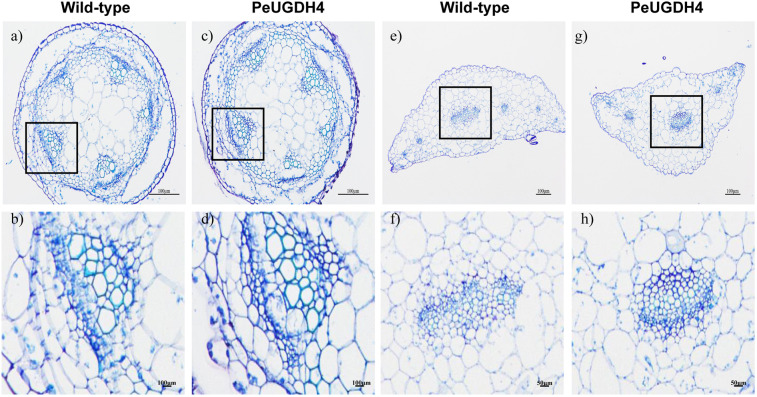
Tissue structure indicated better cell wall development of 40-day-old *PeUGDH4-3* transgenic line than wild-type (WT). Cross sections of stem (**a–d**) and the fifth leaf (**e–h**) after toluidine blue-staining.

To observe cell wall composition more intuitively, paraffin sectioning technique combined with toluidine blue staining was used to make cellulose blue-purple and lignin blue-green. Based on the cell wall content result of WT and transgenic *Arabidopsis*, *PeUGDH4-3* transgenic line was chosen for further paraffin sectioning due to its highest hemicellulose and lignin content. This results showed that in the stem of *A. thaliana*, the number of xylem cells and the content of epidermal cellulose in the *PeUGDH4-3* transgenic plant were higher than those in the wild-type plant and the pith cells were arranged more densely (Fig. 11a–d). In the leaf tissue, the xylem of the vein of *PeUGDH4-3* transgenic plants was closely arranged and the phloem was deeply stained (Fig. 11e–h). These results implied that the *PeUGDH4* gene may play a key role in thickening cell wall structure.

## Discussion

### Characteristics of the PeUGDH gene family

Earlier investigations proved that UGDHs have a central role in the synthesis of many nucleotide sugars, such as the UDP-glucose involved in cell wall biosynthesis, thereby influencing the cell wall structure and strength as well as the development of plant tissues and organs^28,29^. Early in 2002, UGDHs were confirmed as marker enzymes for developing xylem cells from cambial meristems in trees because of their tight correlation with cell division and growth^9^. In the current study, we identified nine putative *UGDH* genes in moso bamboo following a genome-wide analysis. Additionally, there was no major difference in the number of *UGDH* genes between moso bamboo and the other analyzed plant species. For example, *G. max*^13^, *Z. mays*^14^, and *A. thaliana*^8^ carry 10, 2, and 4 *UGDH* genes, respectively, indicating that plant *UGDH* gene families are small.

Because there are only four *AtUGDH* genes in *A. thaliana*, we were unable to further classify the nine putative *PeUGDH* genes into subfamilies based on the *A. thaliana* genes. However, the UGDHs of 11 plant species, including moso bamboo, were included in a phylogenetic analysis, which revealed some clues regarding the evolution of the corresponding genes. Specifically, these plant UGDHs are highly conserved in three groups (Group A: aquatic algae; Group B1: dicotyledons; and Group B2: monocotyledons), implying *UGDH* gene families may have evolved from a common ancestor before the differentiation of aquatic and terrestrial plants. The results of our phylogenetic analysis of moso bamboo evolution is consistent with that of cotton^30^.

A multiple sequence alignment confirmed that all PeUGDHs contain three core domains, with the UDP-glucose/GDP-mannose dehydrogenase domain possibly indispensable for glycometabolism during the formation of the cell wall. Moreover, the functional domains of PeUGDHs, such as MPK3_PHOSPHO_SITE, comprise several phosphorylation sites involved in modifying protein activities to enable the flexible regulation of plant development^31,32^. Furthermore, AtUGDHs reportedly can be phosphorylated at S_393_ by an activated AtMPK3^33^. Phosphorylated AtUGDH3 retains its full enzymatic activity to maintain sufficient amounts of the UDP-sugars needed for cell wall synthesis. In this study, we determined that moso bamboo PeUGDHs also carry a conserved MAP-kinase motif (-P-M-S-P-), suggesting these enzymes may be functionally similar to AtUGDHs when phosphorylated. However, whether phosphorylated PeUGDHs affect cell wall formation will need to be clarified in future studies.

### Evolutionary patterns and structural features of *PeUGDH* genes

Gene duplication events throughout long-term evolution are important for the adaptation of organisms to changing environments^34,35^. On the basis of the Ks values calculated in this study, the homologous *PeUGDH* gene pairs were not the result of whole genome duplications, which would have involved triploid doubling (KS = 1.5~1.8), or modern genome-wide duplications (KS = 0.15~0.3) during the evolution of *PeUGDH* genes^36^. A lack of triploid doubling has resulted in a small *PeUGDH* gene family. According to a previous study, gene duplications contributed to the evolution of genomes and genetic systems^37^. In plants, segmental and tandem duplications occurring most frequently have been important for the expansion of gene families^38^. We determined that eight homologous gene pairs were involved in segmental duplications, whereas two homologous genes located close to each other are tandemly duplicated genes. Accordingly, segmental and tandem duplications are primarily responsible for the expansion of the *PeUGDH* family. Interestingly, in our study, PH02Gene09808.t1 and PH02Gene20841.t1 were clustered separately in C1 in the PeUGDH phylogenetic tree, implying tandem duplications contributed to the development of gene clusters^39,40^. Overall, the duplication events may have contributed to the uneven chromosomal distribution of *PeUGDH* genes during the expansion of the *PeUGDH* gene family.

There is increasing evidence that exon–intron and protein motif sequence structures of gene families may result in differential functions influencing plant growth. Among the *PeUGDH* genes, PH02Gene21682.t1, PH02Gene38725.t1, and PH02Gene21681.t1 in C1 as well as PH02Gene47615.t1 and PH02Gene47617.t1 in C2 share similar exon–intron structures, suggesting the phylogenetic relationships among *PeUGDH* genes are correlated with changes in exon–intron structures. We predicted 10 putative conserved PeUGDH motifs in moso bamboo. Importantly, we noticed that there was a repeating motif phenomenon involving motifs 2 and 10 encoded in *PeUGDH* genes. This phenomenon was also detected for motifs 5 and 20 in the moso bamboo *SBP*-like gene family^41^. The relative lack of difference in 10 conserved motifs implied PeUGDHs may have similar glycometabolic activities during moso bamboo growth. In contrast, the enzymes encoded by the C1 genes PH02Gene09808.t1 and PH02Gene20841.t1, which lack motifs 4 and 10, respectively, may have diverse functions.

### Overexpression of *PeUGDH4* caused changes in cell wall components

Gene expression patterns are highly correlated with gene functions^42^. We observed that 77% of the *PeUGDH* genes were most highly expressed in the stem, followed by the leaf, with the lowest expression levels in the root. Accordingly, the relationship between the gene expression patterns of *PeUGDHs* and cell wall content in three tissues (Fig. 7) implied that most *PeUGDHs* are important for the synthesis of hemicellulose and lignin. Further, several researchers^43^ have confirmed that NtUGDH is the key enzyme of UDP-glucose catalysis on UDP-xylose pathway. And UDP-xylose is also an important precursor of hemicellulose in secondary wall, which is related to the formation of secondary wall.

In this study, overexpressing *PeUGDH4* significantly contributed to increasing content of hemicellulose and soluble sugar in transgenic *Arabidopsis* plants. Also, previous studies have demonstrated that overexpression of *LgUGDH* increases the hemicellulose content and thickens the cell wall in the stem of transgenic *A. thaliana* plants^44^. Similar results were reported in *Zea* plant, a mutation of the *UGDH-A1* gene in *Z. mays* can adversely affect pentosan contents, implying that UGDH were involved in polysaccharide metabolism and cell wall biosynthesis^14^. Herein, overexpression of *PeUGDH4* in *Arabidopsis* increased the lignin content. Moreover, thicker xylem structure were observed through paraffin sectioning of the *PeUGDH4-3* transformed lines (Fig. 11). Related study has also shown that the UGDH gene was not only a key enzyme in the hemicellulose synthesis pathway, but also would affect lignin synthesis. Samac *et al*.^45^ has reported that overexpression of *NtUGDH* in alfalfa resulted in a 15% increase in xylose, which is essential for hemicellulose synthesis, and caused higher Klason lignin content in all transgenic lines than WT plants.

In view of the results presented in this paper, we assumed that PeUGDHs play an essential role in the hemicellulose synthesis involved in cell wall biosynthesis. However, the specific expression mechanism of *PeUGDHs* remains to be determined. Interestingly, the Moso bambo MYB transcription factor has been identified to be involved in the regulation of cellulose and hemicellulose synthesis and are also related to the metabolism of lignin^26^. In the future, on behalf of the *PeUGDH4* promoter sequence (we have cloned), key transcription factors binding to the cis-acting elements upstream of *PeUGDH4* will be isolated and *PeUGDH4* transcriptional regulation mechanism will be revealed. Elucidation of the molecular modules controlling bamboo cell wall formation should contribute to bamboo research and breeding.

## Methods

### Plant growth conditions

Fresh and plump *P. edulis* fruits were analyzed in this study. After removing the seed glumes, the seeds were thoroughly washed and soaked in a solution comprising 200 mg/L gibberellin for 2 h to induce germination. The resulting seedlings were grown in a growth chamber at 28 °C and 80% relative humidity under long-day conditions (16-h light/8-h dark) for 100 days, after which gene expression levels were determined for various tissues. Specifically, young leaves, the main stem and underground root were collected for an RNA extraction (Fig. S1).

### Database search for moso bamboo *PeUGDH* genes

The OsUGDH protein sequences obtained from the Phytozome database (https://phytozome.jgi.doe.gov/pz/portal.htm) were used as queries to screen for *PeUGDH* genes in the Bamboo database (http://parrot.genomics.cn) with the BLAST algorithm. The identified sequences were aligned with the Clustal W 2.1 program, after which redundant sequences were removed^46^ (Fasta S1, S2). The proteins encoded by the putative *PeUGDH* genes were analyzed with the Pfam (http://pfam.xfam.org/) and National Center for Biotechnology Information CD-search (https://www.ncbi.nlm.nih.gov/Structure/cdd/wrpsb.cgi) programs to determine whether they contain specific conserved UGDH domains. Additionally, *PeUGDH* gene structures were obtained with the Gene Structure Display Server (http://gsds.cbi.pku.edu.cn/)^47^. Basic physical and chemical parameters of the PeUGDH proteins were predicted with the ExPASy online tool^48^ (http://au.expasy.org/tools /pi_tool.html).

### PeUGDH protein alignment and phylogenetic analysis

The Phytozome database was screened for the amino acid sequences of UGDHs from the following 10 species: *G. max*, *Oryza sativa*, *Zea mays*, *Solanum lycopersicum*, *Ananas comosus*, *Volvox carteri*, *Chlamydomonas reinhardtii*, *Populus trichocarpa*, *A. thaliana*, and *Brachypodium distachyon*. To examine the phylogenetic relationships among the UGDHs from moso bamboo and other plants, a multiple sequence alignment of nine PeUGDH proteins and a phylogenetic analysis involving all UGDHs were completed. Multiple sequence alignments were analyzed with the Clustal W 2.1 program and the phylogenetic analysis was conducted according to the maximum likelihood method (1,000 bootstrap replicates) of the MEGA 7.0 software. Functional sites were identified based on the Prosite database.

### Chromosomal localization, duplication analysis, and Ka/Ks calculation

The nine *PeUGDH* genes were localized to three chromosomes based on the genome annotation file (gff file) from the moso bamboo database. We focused on possible segmental and tandem duplications. The MCScanX software was used to identify potential duplication events in moso bamboo. Additionally, using the moso bamboo gff file, the tandemly duplicated *PeUGDH* genes were identified by checking whether candidate genes on individual chromosomes were within 50 kb of each other^49^. Homologous pairs of *UGDH* genes were obtained with Clustal X2.1 and used to calculate the non-synonymous substitution (Ka) and synonymous substitution (Ks) rates with KaKs Calculator 2.0. The Ks value can be used to calculate the divergence time (T)^50^. Specifically, because the special synonymous substitution rate (λ) for moso bamboo is 6.5 × 10^−9^, the divergence time was calculated with the following equation: T = Ks/2λ^51^.

### Protein structure analysis

Protein models of the homologous PeUGDH proteins were predicted with the Phyre^2^ online program (http://www.sbg.bio.ic.ac.uk/phyre2/html/page.cgi?id=index)^24^. Additionally, the online MEME tool was used to identify conserved motifs in the PeUGDH proteins (http://meme-suite.org/tools/meme), with the number of motifs set to 10.

### RNA isolation and quantitative real-time PCR

The quantitative real-time (qRT)-PCR primers listed in Table S1 were designed with the NCBI Primer-blast online tool. Total RNA was isolated from moso bamboo root, stem and leaf with the RNAprep Pure Plant Kit (TransGen Biotech, China). The purified RNA was used as the template to synthesize the first-strand cDNA with the QuantScript RT Kit (TIANGEN, China). The *TIP41* gene (tonoplast intrinsic protein 41) was selected as a reference control because it is stably expressed^52^. The qRT-PCR assay was conducted with the fluorescent dye SYBR Green (TIANGEN, China) and the Bio-Rad CFX96 system, with conditions recommended by the manufacturer. Three biological replicates were used to calculate the mean expression values and standard error of the mean. Relative gene expression levels were determined based on the 2^−ΔΔCt^ method^53^.

### PeUGDH gene expression and cell wall contents in different tissues

The leaf, root and stem of 100-day-old moso bamboo plants were collected as experimental materials. A comprehensive expression profile for each *PeUGDH* gene was generated, with *TIP41* as a reference control.

Additionally, the cellulose, hemicellulose, and lignin contents, which are the main cell wall components, were measured with three commercial kits from KEMING company of China (item numbers CLL-2-Y, BXW-2-G, and MZS-2, respectively).

### Gene cloning, plasmid construction and subcellular localization analysis

Due to the fact that expression was up-regulated during the growth of bamboo shoots and may be as a key gene of *PeUGDHs* in the formation of cell wall hemicellulos, PH02Gene21682 was selected to gain further research. Then, we named PH02Gene21682 as *PeUGDH4* according to its molecular weight. The sequence of *PeUGDH4* was amplified with sense primer (5′-GGGGTACCATGGTGAAGATCTGCTGC-3′) and antisense primer (5′-GCTCTAGAAGCAACCGCGGGCATATC-3′) (restriction endonuclease *KpnI* and *XbaI* were underlined) using cDNA of bamboo stem as the template. The sequence of *Atactin2* was amplified with sense primer (5′-TGGTGTCATGGTTGGGATG-3′) and antisense primer (5′-CACCACTGAGCACAATGTTAC-3′). The PCR program was as follows: predenaturing for 94 °C for 5 min; followed by 35 cycles of 94 °C for 30 s, 60 °C for 30 s and at 72 °C for 50 s, with 72 °C for 10 min for a final extension. After that, the PCR product was cloned into the reconstructed expression vector pCAMBIA2300 containing a green fluorescent protein (*GFP*) gene which was controlled by the CaMV35S promoter (Fig. S2). Constructs were transformed into the *Agrobacterium tumefaciens* strain GV3101 by liquid N2 freeze-thaw method^54^ and the *Arabidopsis* plants were transformed by the floral dip method via *Agrobacterium*-mediated transformation procedure. Taq polymerase were purchased from TransGen Biotech company (China). The correct *Agrobacterium* strains were cultured at 28 °C for 48 h in a ratio of 1:1000, and the suspensions were injected into the leaves of *Nicotiana tabacum* according to the method described by Sainsbury *et al*.^55^. Therefore, GFP fluorescence was observed with A1R-si laser scan confocal microscope (Nikon, Japan)^56^.

### Selection and analysis cell wall content of transgenic *Arabidopsis*

The transgenic *Arabidopsis* plants were selected on Murashige and Skoog (MS) solid medium containing 100 mg/L kanamycin for one week at 25 °C in the culture room. And then seedlings were transferred to the soil mix {peat, perlite and vermiculite (2:1:2)} and grown in greenhouse at 25 °C. T1 seeds were harvested and germinated on medium supplemented with 100 mg/L kanamycin for selection of transgenic plants. CTAB protocol was used to extract total DNA from non-transgenic and three T1 transgenic lines (*PeUGDH4*-*1*, *PeUGDH4*-*2* and*PeUGDH4*-*3*) which was confirmed by PCR technique using above primers and program. Then, these stable heritable transgenic *Arabidopsis* lines was cultured to detect *PeUGDH4* gene expression at translation levels. Additionally, the content of soluble sugar, cellulose, hemicellulose and lignin which were the main cell wall components of the fifth rosette leaf of 40-day-old *A. thaliana* plants was measured with three commercial kits (item numbers A145-1-1, CLL-2-Y, BXW-2-G and MZS-2-G respectively) (KEMING, China). In detail, the cellulose monomer β-glucose could produce β-furfural substance under the action of sulfuric acid, which produced color reaction with anthrone dehydration and condensation, this absorbance value was read at 620 nm. Hemicellulose was converted into reducing sugar after sulfuric acid treatment, which absorbance value was measured at 540 nm. The phenolic hydroxyl group in lignin had a characteristic absorption peak at the wavelength of 280 nm after acetylation, and this absorbance was positively correlated with the lignin content. The content of soluble sugar can be calculated by anthrone colorimetry, which could be combined with soluble monosaccharides, oligosaccharides and polysaccharides. The absorbance is determined at 620 nm, and the soluble sugar content is calculated in 1.17×(ΔA+ 0.07) /W.

### Paraffin sectioning and histological staining

Midribs from the fifth leaf and stem tissue taken from ~2 cm from the bottom of *Arabidopsis* plant were collected from 40-day-old *PeUGDH4* transgenic and wild-type *Arabidopsis* plants. These tissues were fixed in the solution including 75% ethanol and 25% acetic acid. The procedures of paraffin sectioning as described by Cai and Lashbrook^57^. After that, sections were dewaxing for 30 min and stained for 2–3 min in 0.02% toluidine blue (Sigma, USA), imaged using an Olympus BX-61 light microscope (Olympus Co.).

### Statistical analyses

Experiments were conducted in three biological replicates with each replicate consisting of three plants. All data were analyzed using SPSS (version 19.0; IBM Corp., Armonk, NY, USA) and were expressed as the mean ± SD (standard deviations). Multigroup comparisons of the means were carried out by one-way analysis of variance (ANOVA) test with post hoc contrasts by Student–Newman–Keuls test. The statistical significance for all tests was set at *P* < 0.05. All charts were plotted using Microsoft Excel.

## Supplementary information

Supplementary information.
