# Supplementary material for: Genome-wide identification and characterization of UDP-glucose dehydrogenase family genes in moso bamboo and functional analysis of PeUGDH4 in hemicellulose synthesis
Source: Sci Rep. 2020 Jun 23;10:10124. doi: 10.1038/s41598-020-67227-8 (PMC7311537; doi:10.1038/s41598-020-67227-8)
Supplement: Supplementary file 1 — Supplementary information. [file 41598_2020_67227_MOESM1_ESM.zip › Supplementary Information/supplementary file (figures and tables).doc]

***Supplementary information***

Genome-wide identification and characterization of UDP-glucose dehydrogenase family genes in moso bamboo and functional analysis of *PeUGDH4* in hemicellulose synthesis

**Authors:**

Ying Yang^1,2^, Lan Kang^1,2^, Ruihua Wu^1,2^, Yuzhen Chen^1,2^, Cunfu Lu^1,2^*

[yangying1994@bjfu.edu.cn (Y.Y.)](mailto:wuruihua@bjfu.edu.cn (R.W.)); [KangLan@bjfu.edu.cn](mailto:syrshiyanrong@hotmail.com) (L.K.); [wuruihua@bjfu.edu.cn (R.W.)](mailto:wuruihua@bjfu.edu.cn (R.W.)); chenyuzhen@bjfu.edu.cn (Y.C.)

***** Correspondence: [lucunfu@bjfu.edu.cn](mailto:lucunfu@bjfu.edu.cn) (C. L.)

**Affiliations：**

**^1^**Beijing Advance Innovation Center for Tree Breeding by Molecular Design, Beijing Forestry University, Beijing 100083, China;

**^2^**College of Biological Sciences and Technology, Beijing Forestry University, Beijing 100083, China;

**Supplementary Information**

Figure. S1. Three moso bamboo tissues analyzed by qRT-PCR.

Figure. S2. Molecular identification of *PeUGDH4*-transformed *Arabidopsis* lines.

Figure. S3. Sequence analysis of PeUGDH4.

Table S1. Details regarding the qRT-PCR primers for nine *PeUGDH* genes.

Table S2. MEME motif sequences and lengths of moso bamboo PeUGDH proteins.

Table S3. The microarray expression data of nine *PeUGDH* genes in moso bamboo.

**Figure S1.** Three moso bamboo tissues analyzed by qRT-PCR.

**Figure S2.** Molecular identification of *PeUGDH4*-transformed *Arabidopsis* lines. **a)** Schematic representation of the pCAMBIA2300-*PeUGDH4*-GFP construct used for *Arabidopsis* transformation.**b)** RT-PCR analysis of the WT and *PeUGDH4* transgene *Arbidopsis* lines with an internal control (*AtActin2* gene). **c)** RT-PCR analysis of the WT and *PeUGDH4* transgene *Arbidopsis* lines with the primer of *PeUGDH4* gene.

**Figure S3.** Sequence analysis of PeUGDH4. **a)** The signal peptide in the predicted amino residues of PeUGDH4. **b)**The transmembrane structure analysis in the predicted amino residues of PeUGDH4.

**Table S1.** Details regarding the qRT-PCR primers for nine PeUGDH genes.

| **Genes** | **Primers used in qRT-PCR(5′→3′)** | |
| --- | --- | --- |
| **PH02Gene21682.t1** | Forwards | ACGAGTTTTCCCTCAGGCAT |
|  | Reverse | ACCTTCGGATGGTCCTCAAG |
| **PH02Gene38725.t1** | Forwards | TTTTTGCAGGAGGCACTACGA |
|  | Reverse | CAGACAGCGTTGCAAACAAGA |
| **PH02Gene21681.t1** | Forwards | CACAAGTCACATCGGAAGCG |
|  | Reverse | AAACTCGTAGCGTCTCCTGC |
| **PH02Gene29082.t1** | Forwards | CCACCCCTTACCTTTCACCTG |
|  | Reverse | ACGATGCAACAAAATACACTCCA |
| **PH02Gene19803.t1** | Forwards | GCGTTCGCACCTCCCTATTT |
|  | Reverse | GCGTTCGCACCTCCCTATTT |
| **PH02Gene09808.t1** | Forwards | AAGTTCGACTGGGATCACCC |
|  | Reverse | GTTCCTGAACACGTCCCACT |
| **PH02Gene20841.t1** | Forwards | CAAATCCAGCGTGACCTTGC |
|  | Reverse | ATCCTTGGCTGCCTCATACG |
| **PH02Gene47615.t1** | Forwards | TCAGCATGTACGACCCGC |
|  | Reverse | GATTCTCCGGTACTCGAGCG |
| **PH02Gene47617.t1** | Forwards | GTTCAGGACGCTCGACTACC |
|  | Reverse | CTATCTCCCTGAGCTTGCTGG |

**Table S2.** MEME motif sequences and lengths of moso bamboo PeUGDH proteins.

| **Motif** | **Width** | **Best possible match** |
| --- | --- | --- |
| 1 | 50 | CFQKDILNLVYICECNGLPEVANYWKQVIKINDYQKSRFVNRVVSSMFNT |
| 2 | 50 | KZCRGRNLFFSTDVEKHVAEADIIFVSVNTPTKTRGLGAGKAADLTYWES |
| 3 | 50 | LKDVYAHWVPEDRIITTNLWSAELSKLAANAFLAQRISSVNALSALCEAT |
| 4 | 50 | KTAEAIEKILTHNSKGINFQILSNPEFLAEGTAIQDLFKPDRVLIGGRET |
| 5 | 50 | HGVCILTEWDEFKTLDYKKIYDNMQKPAFVFDGRNIVDAEKLREIGFIVY |
| 6 | 50 | ICCIGAGYVGGPTMAVIALKCPAIEVVVVDISKPRIEAWNSDQLPIYEPG |
| 7 | 50 | TRETPAIDVCKGLLGDKAKISIYDPQVTEDQIQRDLAMNKFDWDHPMHLQ |
| 8 | 29 | NVSEVAYAVGKDSRIGPKFLNASVGFGGS |
| 9 | 15 | SKSDKIVVEKSTVPV |
| 10 | 15 | KIAVLGFAFKKDTGD |

**Table S3.** The microarray expression data of nine *PeUGDH* genes in moso bamboo.

| **gene ID** | **leaf** | **stem** | **root** |
| --- | --- | --- | --- |
| PH02Gene21682.t1 | 1.58437 | 1.57869 | 0.22363 |
| PH02Gene38725.t1 | 1.00000 | 0.15568 | 0.11723 |
| PH02Gene21682.t1 | 1.05455 | 2.17400 | 0.16227 |
| PH02Gene29081.t1 | 0.00206 | 0.79878 | 0.17773 |
| PH02Gene19803.t1 | 0.29698 | 2.17400 | 0.01034 |
| PH02Gene09808.t1 | 1.48516 | 2.17400 | 0.08283 |
| PH02Gene20841.t1 | 1.00000 | 0.10156 | 0.10839 |
| PH02Gene47615.t1 | 0.04159 | 1.57869 | 0.42589 |
| PH02Gene47617.t1 | 0.33409 | 1.57869 | 0.18112 |
